# Simulation of Vibrational Circular Dichroism Spectra Using Second-Order Møller–Plesset Perturbation Theory and Configuration Interaction Doubles

**DOI:** 10.1021/acs.jctc.4c00747

**Published:** 2024-08-13

**Authors:** Brendan M. Shumberger, T. Daniel Crawford

**Affiliations:** Department of Chemistry, Virginia Tech, Blacksburg, Virginia 24061, United States

## Abstract

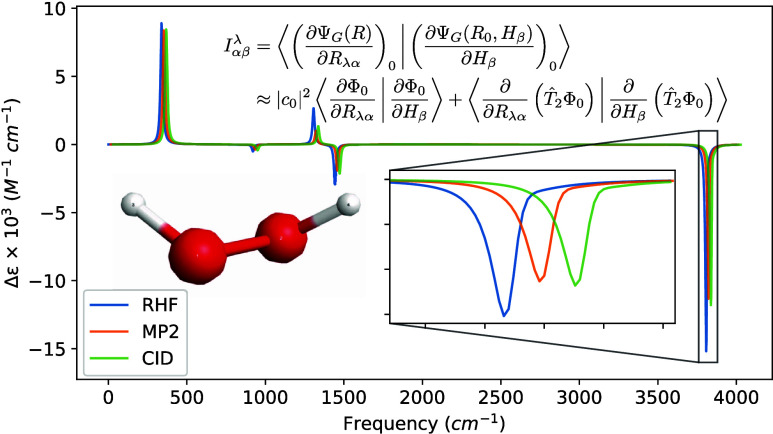

We present the first single-reference calculations of the atomic axial tensors (AATs) with wave function-based methods including dynamic electron correlation effects using second-order Møller–Plesset perturbation theory (MP2) and configuration interaction doubles (CID). Our implementation involves computing the overlap of numerical derivatives of the correlated wave functions with respect to both nuclear displacement coordinates and the external magnetic field. Out test set included three small molecules, including the axially chiral hydrogen molecule dimer and (*P*)-hydrogen peroxide, and the achiral H_2_O. For our molecular test set, we observed deviations of the AATs for MP2 and CID from that of the Hartree–Fock (HF) method upward of 49%, varying with the choice of basis set. For (*P*)-hydrogen peroxide, electron correlation effects on the vibrational circular dichroism (VCD) rotatory strengths and corresponding spectra were particularly significant, with maximum deviations of the rotatory strengths of 62 and 49% for MP2 and CID, respectively, using our largest basis set. The inclusion of dynamic electron correlation to the computation of the AATs can have a significant impact on the resulting rotatory strengths and VCD spectra.

## 1 Introduction

Vibrational circular dichroism (VCD) is one of the unique spectroscopies whose experimental discovery was preceded by theoretical predictions. The first successful attempt to simulate Cotton effects in the infrared region of the electromagnetic spectrum was reported by Deutsche and Moscowitz in 1968 on a model helical polymer,^1,2^ though the expressions they derived were not generally applicable. Experimental measurements of VCD rotatory strengths followed in 1974 in neat solutions of (*S*)-(+)- and (*R*)-(−)-2,2,2-trifluoro-1-phenylethanol^3^ for which the only observed vibrational modes were those of the C–H stretching motions on the chiral carbon. During this same period, a profusion of *ad hoc* models for predicting VCD spectra were developed, including the coupled oscillator model^4^, the fixed partial charge model^5^, the localized molecular orbital model^6^, the charge flow model^7^, the ring current model^8,9^, the atomic polar tensor model^10^ and others.^11−13^ The principal theoretical challenge that spurred these new models is the fact that the VCD rotatory strength of a given vibrational transition is zero in the Born–Oppenheimer approximation. In particular, the magnetic-dipole vibrational transition moment, whose product with the corresponding electric-dipole transition moment yields the rotatory strength, vanishes for adiabatic wave functions. This unphysical behavior proved highly nontrivial to overcome through a generalized, first-principles approach, and it was found that these heuristic models exhibited severe limitations that precluded their widespread applicability.^14,15^

In 1983, Nafie and Freedman put forth the vibronic coupling theory,^16^ which superseded the limitations of the BO approximation via introduction of a nuclear perturbation to the adiabatic wave function. This approach led to a sum-overstates expression that the authors were able to reduce to one involving only the ground state wave function using an average-energy approximation. In 1985, Stephens introduced the first fully ground-state formulation of VCD rotatory strengths.^17^ By introducing first-order perturbations of the ground-state wave function with respect to the external magnetic field and a nuclear displacement, and then equating these expressions with the first-order Taylor expansions in the same variables, he was able to reduce the resulting expression solely to an overlap of derivatives of the ground-state wave function, known as the atomic axial tensor (AAT). Much more recently, a response formalism of VCD was proposed by Coriani et al.^18^ which has the advantage that its implementation into Kohn–Sham density functional theory (KS-DFT) avoided the need to solve the response functions for the 3*N* geometric displacements, thereby reducing the computational cost. To date, Stephens’s formulation has been the most widely implemented approach to calculating the magnetic dipole vibrational transition moment required to compute VCD.

The first implementation of the atomic axial tensors (AATs) of Stephens’s VCD formulation was reported at the Hartree–Fock level by Lowe, Segal, and Stephens using numerical differentiation of single-determinant wave functions with respect to nuclear coordinates and external magnetic fields.^15,19^ Shortly thereafter Amos, Handy, Jalkanan, and Stephens,^20^ reported the first analytic evaluation of Hartree–Fock AATs based on solutions to the nuclear and magnetic-field coupled-perturbed Hartree–Fock (CPHF) equations, validated by the corresponding finite-difference approach. The analytic approach is substantially more computationally efficient because it not only avoids the 6*N* + 6 calculations for the nuclear displacements and magnetic-field coordinates, but it also avoids the complex arithmetic required for finite magnetic fields.

In 1993, Bak et al.^21^ reported the anaytic implementation of AATs at the multiconfigurational self-consistent field (MCSCF) level of theory. The authors used an orbital rotation formulation to obtain an equation for the AATs in terms of density matrices, integrals, derivatives of molecular orbital coefficients, and derivatives of configuration state coefficients for which they solved for the coefficient derivatives using the response formalism developed by Helgaker and Jørgensen.^22^ In addition, they used gauge-including atomic orbitals (GIAOs) to circumnavigate the gauge-origin problems that typically plague magnetic-field dependent properties. Though they made no direct comparison to experiment, the authors made note that the correlation effects included by the MCSCF calculation on NHDT, the isotopomer of ammonia, were significant. In 1994, Stephens et al.^23^ reported the first application of Møller–Plesset (MP) perturbation theory and Kohn–Sham density-functional theory (KS-DFT) to VCD spectra. However, only the harmonic force fields were computed at these levels, while the AATs were still obtained using Hartree–Fock. (We note that this was also the first paper to define the B3LYP exchange-correlation functional, though that was not the focus of the work.)

In 1996, Cheeseman and co-workers^24^ carried out the first fully analytic DFT-based AAT implementation, including the use of GIAOs to ensure origin-invariant rotatory strengths. The results from this implementation compared well with experiment for *trans*-2,3 *d*_2_-oxirane, though the authors noted that the accuracy of the DFT results depend on the density functional adopted, an observation that has been echoed in the literature for a number of VCD applications.^25−28^

Here we present the first calculations of VCD AAT rotatory strengths using dynamically correlated wave function methods. In particular, we have implemented finite-difference gradients of first-order MP and configuration interaction doubles (CID) wave functions with respect to nuclear coordinates and external magnetic fields and combined these to obtain the resulting AATs. We have applied these methods to a number of small-molecule test cases, including hydrogen peroxide.

## 2 Theory

### 2.1 Vibrational Circular Dichroism

The simulation of VCD spectra requires calculation of the differential molar extinction coefficient, Δϵ^29^
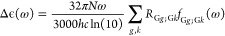
1where *h* is Planck’s constant, *c* is the speed of light, and ω is the harmonic angular frequency. The functions *f*_G*g*;G*k*_(ω) and *R*_G*g*;G*k*_ are the line shape function and rotational strength, respectively, both of which are associated with the *g* → *k* transition of a given vibrational mode within the electronic ground state, G. The line shape function, or spectral broadening function, for vibrational transitions is typically modeled by a Lorentzian function
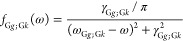
2where γ_G*g*;G*k*_ is half of the peak width at half the peak height for a given transition with frequency, ω_G*g*;G*k*_. The primary quantity of interest, the rotatory strength, is
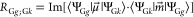
3where μ⃗ and *m⃗* are the electric- and magnetic-dipole operators, respectively. In the vibrational harmonic approximation, the electric-dipole transition moment of the *i* normal mode is given by the ν = 0 → 1 transition^30^
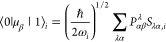
4where ω_*i*_ is the harmonic angular frequency associated with the normal mode, *P*_αβ_^λ^ are the atomic polar tensors (APTs), and *S*_λα,*i*_ is the normal coordinate transformation matrix from Cartesian nuclear displacements to mass-weighted normal mode displacements. In this notation, β denotes a particular Cartesian direction of the external electric field, λ indexes the nuclei, and α is a Cartesian coordinate of the λ-th nucleus. The APTs are typically computed using the electrical harmonic approximation, i.e., as derivatives of the molecular dipole moment with respect to nuclear displacements
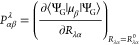
5where ⟨Ψ_G_|μ_β_|Ψ_G_⟩ is the electric-dipole moment expectation value in the electronic ground state, and *R*_λα_^0^ denotes the equilibrium/reference geometry.

Similarly, the magnetic-dipole transition moment is expressed as
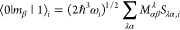
6where *M*_αβ_^λ^ is the AAT, λ and α have the same meaning as in eq 5, and β denotes a particular Cartesian direction of the external magnetic field. The AAT can be separated into its nuclear and electronic components as
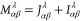
7The nuclear contribution is given by
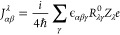
8where ϵ_αβγ_ is the three-dimensional Levi-Civita tensor, *R*_λγ_^0^ is the γ-th equilibrium Cartesian coordinate of the λ-th nucleus, and *Z*_λ_*e* is the charge of the λ-th nucleus.

The primary focus of the present work is the electronic contribution to the AAT, which, in Stephens’s formulation is^17^
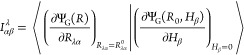
9Thus, the challenge is to compute the overlap of two derivatives of the ground-state wave function: one with respect to nuclear displacements, *R*_λα_, and one with respect to the external magnetic field, *H*_β_, both evaluated at the equilibrium geometry and at zero-field.

### 2.2 Electronic AATs within the MP2 and CID Wave Function Approximations

In both MP2 and CID, the electronic wave functions are of the form
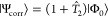
10where Ψ_corr_ is the correlated wave function (MP2 or CID), *T̂*_2_ is the double-excitation operator, and Φ_0_ is the reference Hartree–Fock (HF) wave function. In spin–orbital notation, the *T̂*_2_ operator is given by
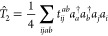
11with *i*, *j*, *k*, and *l* to represent occupied orbitals and *a*, *b*, *c*, and *d* to virtual orbitals. The cluster amplitudes, *t*_*ij*_^*ab*^ in eqs 10 and 11 are obtained from the first-order wave function equation for MP2
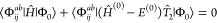
12where |Φ_*ij*_^*ab*^⟩ is a doubly excited determinant, *Ĥ* is the electronic Hamiltonian, *Ĥ*^(0)^ is the Fock operator, and *E*^(0)^ is the sum of the occupied orbital energies. For CID, the amplitudes are obtained from the projection of the CI Schrödinger equation onto the doubly excited determinants
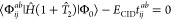
13Stephens’s construction of the AAT in eq 9 requires that the ground-state wave function is fully normalized, but the above expressions assume intermediate normalization. Thus, after the amplitudes have been computed, they must be renormalized such that
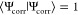
14

Incorporation of the normalized form of eq 10 into eq 9 yields four terms
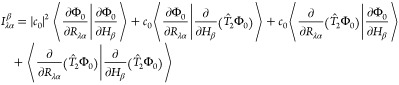
15where *c*_0_ denotes the coefficient of Φ_0_ in the fully normalized wave function. As shown by Pearce,^31^ the second and third terms on the right-hand side are equal in magnitude, but opposite in sign, and thus exactly cancel. Following Stephens and co-workers,^15,19,20^ we have computed the above derivatives using a finite-difference approach in which the HF and MP2/CID wave function is computed after the displacement of a given nuclear coordinate or by the addition of a weak magnetic field in a chosen direction to the Hamiltonian. For each combination of displaced wave functions, their overlap is obtained by multiplying the overlap of the corresponding determinants with the associated *T̂*_2_ amplitudes and/or *c*_0_ coefficients. However, because the displaced wave functions are in different MO bases, the overlaps between Slater determinants are computed by taking the determinant of the MO-basis overlap matrix of the orbitals comprising the bra and ket determinants. For example, the first term in eq 15 involves derivatives between HF determinants such that the finite difference expansion is
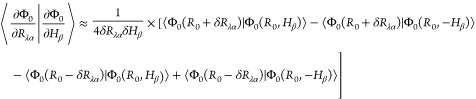
16where δ*R*_λα_ and δ*H*_β_ are the geometric displacements and magnetic field perturbations, respectively. Using the determinant product rule, the terms in eq 16 can be obtained from
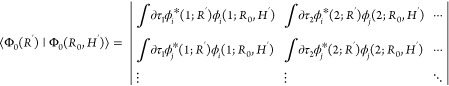
17with *R*′ = *R*_0_ ± δ*R*_λα_ and *H*′ = ± δ*H*_β_ from which the determinant is taken from only the set of occupied orbitals in the overlap matrix. For excited determinants, this matrix is obtained by swapping the appropriate rows (bra) and/or columns (ket) corresponding to the relevant occupied and virtual spin–orbitals.

As pointed out by Stephens et al.,^15,19^ one must carefully account for the phases of the displaced wave functions in order to obtain physically correct values of the wave function overlaps in the finite-difference scheme. For the nuclear coordinate displacements, the HF and correlated wave functions remain real, and so the phase factors are associated only with the signs of the individual HF MOs. For magnetic-field displacements, the wave functions are complex, and thus the MO coefficients and cluster amplitudes are also complex. Following Stephens,^19^ the presence of a magnetic-field perturbation in the Hamiltonian yields a complex phase factor between two normalized MOs
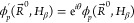
18where ϕ_*p*_(*R̂*^0^, *H*_β_) is the MO that reduces to ϕ_*p*_(*R⃗*^0^) in the absence of the field. For weak fields, the overlap of ϕ_p_^′^(*R⃗*^0^, *H*_β_) with the zero-field MO is
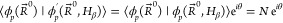
19where *N* is the (real) normalization constant of ϕ_*p*_^′^(*R⃗*^0^, *H*_β_) through second order in the field. Squaring this overlap yields the square of *N*
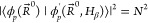
20which allows us to obtain the value of e^*i*θ^ from the overlap in eq 19 and correct the phase of ϕ_*p*_^′^(*R⃗*^0^, *H*_β_). (Note that this same approach also serves to correct the phases on the MOs for real perturbations, in which case the phase factor simplifies to ±1.) Once the phases of the MOs have been defined, the phases of the amplitudes are also defined because of the projection-based approach we use in eqs 12 and 13.

## 3 Computational Details

We have implemented the finite-difference scheme described above in an open-source Python code, MagPy,^32^ which uses the form given in eq 15 to compute the AAT. Given that MP2 and CID AATs have not yet been reported in the literature, we have tested and validated this code in several ways: (1) we have developed a second, independent Python implementation that splits the derivatives of the cluster amplitudes and the Slater determinants into separate finite-difference calculations; (2) we have derived and coded both spin–orbital and spin-adapted (spatial orbital) forms for both MP2 and CID AATs; (3) we have compared our (non-GIAO) Hartree–Fock AATs to those produced by the DALTON code^33^ for all test cases; (4) we have computed all four contributions appearing in eq 15 to confirm that the second and third terms cancel. Both of these implementations use the Psi4 quantum chemistry package^34^ to provide the necessary Hamiltonian, electric-/magnetic-dipole, and mixed-basis-set overlap integrals.

To analyze the impact of dynamic electron correlation effects on the electronic AATs, we have selected three molecular test cases, a hydrogen molecule dimer, water, and (*P*)-hydrogen peroxide, as well as numerous basis sets: STO-3G, 6-31G, 6-31G(d), cc-pVDZ, and cc-pVTZ, depending on the size of the molecule.^35−42^ The hydrogen molecule dimer is included because it is the smallest possible chiral system, which has been used in previous studies as representative of the beginning of a helical hydrogen system for benchmarking calculations of optical rotation.^43^ Our chosen (nonoptimized) structure of the hydrogen molecule dimer has an intramolecular H–H distance of 0.75 Å, an intermolecular H–H distance on the helical backbone of 1.0 Å, and a + 60° dihedral angle. The geometries for water and (*P*)-hydrogen peroxide were optimized at the MP2/cc-pVDZ level of theory using the CFOUR quantum chemistry package.^44^ [Geometries of all three test molecules are given in the Supporting Information (SI).] The 1*s* core orbitals of the oxygen atoms were frozen in all MP2 and CID calculations on water and (*P*)-hydrogen peroxide.

We report rotatory strengths and corresponding VCD spectra only for (*P*)-hydrogen peroxide, because it is the only chiral compound in our test set for which the equilibrium geometry remains chiral (for basis sets that include polarization functions). Inherent performance limitations of Python as well as the cost of evaluating the overlap of two doubly excited determinants in different bases in eq 15 precluded application of the code to larger molecules. In order to focus on the impact of dynamic electron correlation effects on the spectra, we used a common geometry and harmonic force field (both computed at the MP2/cc-pVDZ level of theory) for all basis sets. In addition, because we do not include GIAOs in our implementation, the VCD rotatory strengths we report are origin dependent. As such, keeping the geometry fixed reduces the impact that reoptimizing the molecular structure for each basis set has on the resultant AATs.

## 4 Results and Discussion

### 4.1 Atomic Axial Tensors

In order to provide a simple quantitative comparison of these tensors, we report correlation minimum/maximum percent changes, which are obtained by dividing each element of a given correlated AAT by its Hartree–Fock counterpart, subtracting 1.0 from each element and converting to a percentage. This is intended to provide an estimate of magnitude of electron correlation effects on the AAT elements. The corresponding impact on VCD rotary strengths will be discussed in the next section.

The AATs computed for the hydrogen molecule dimer at the HF, MP2, and CID levels of theory are given in Table 1 using cc-pVDZ basis set. (The results from the STO-3G, 6-31G, and cc-pVTZ basis sets are provided in the SI). The minimum/maximum percentages of the HF AATs for MP2 are 98.5/99.0% for STO-3G, 97.7/102.4% for 6-31G, 95.6/101.6% for cc-pVDZ, and 97.6/106.2% for cc-pVTZ. For CID, the minimum/maximum percentages are 96.2/98.1% for STO-3G, 95.2/107.5% for 6-31G, 83.5/105.6% for cc-pVDZ, and 92.6/121.3% for cc-pVTZ. As is clear, the gap between the minimum and maximum percentages increases for both MP2 and CID as the basis set size grows. Additionally, for each basis set, the difference between the maximum and minimum values is always greater for CID over MP2. This difference reaches a maximum of 28.8% for the hydrogen molecule dimer using CID with the cc-pVTZ basis (see Table S7 of the SI).

**Table 1:** Electronic HF, MP2, and CID AATs (a.u.) for the Hydrogen Molecule Dimer Using the cc-pVDZ Basis

	HF			MP2			CID		
	*B*_*x*_	*B*_*y*_	*B*_*z*_	*B*_*x*_	*B*_*y*_	*B*_*z*_	*B*_*x*_	*B*_*y*_	*B*_*z*_
*H*_1*x*_	–0.078893	–0.008996	0.364298	–0.078875	–0.008600	0.362977	–0.078636	–0.007509	0.362176
*H*_1*y*_	0.031734	0.009958	0.073418	0.031340	0.009951	0.073174	0.030302	0.009932	0.073471
*H*_1*z*_	–0.529172	–0.138045	0.085432	–0.527501	–0.137850	0.085348	–0.525801	–0.138038	0.085000
*H*_2*x*_	–0.053675	–0.025007	0.342479	–0.053706	–0.025413	0.341287	–0.053566	–0.026407	0.340754
*H*_2*y*_	–0.024080	–0.008875	0.109311	–0.023638	–0.008864	0.108857	–0.022607	–0.008877	0.108197
*H*_2*z*_	–0.523197	–0.123777	0.078075	–0.521843	–0.123201	0.078051	–0.520677	–0.122274	0.077854
*H*_3*x*_	–0.053675	–0.025007	–0.342479	–0.053706	–0.025413	–0.341287	–0.053566	–0.026407	–0.340754
*H*_3*y*_	–0.024080	–0.008875	–0.109311	–0.023638	–0.008864	–0.108857	–0.022607	–0.008877	–0.108197
*H*_3*z*_	0.523197	0.123777	0.078075	0.521843	0.123201	0.078051	0.520677	0.122274	0.077854
*H*_4*x*_	–0.078893	–0.008996	–0.364298	–0.078875	–0.008600	–0.362977	–0.078636	–0.007509	–0.362176
*H*_4*y*_	0.031734	0.009958	–0.073418	0.031340	0.009951	–0.073174	0.030302	0.009932	–0.073471
*H*_4*z*_	0.529172	0.138045	0.085432	0.527501	0.137850	0.085348	0.525801	0.138038	0.085000

The water molecule provides a reasonable test case for investigating the effect of electron correlation on the AATs, just as it has for a range of other properties. Although the molecule is achiral, it contains multiple nonzero AAT elements for comparison between methods. AATs for water are given in Table 2 for HF, MP2, and CID, again using the cc-pVDZ basis set. (Results obtained for the remaining basis sets are provided in the SI). The *C*_2*v*_ symmetry, with the molecule lying the *yz*-plane and the *C*_2_ axis along the *z*-axis, limits the number of nonzero tensor components. In particular, only elements for which the direct product of the irreducible representations (irreps) of the *R*_λα_ displacement and the *H*_β_ field component contains the totally symmetric irrep can be nonvanishing. The *H*_β_ terms transform as rotations about the three Cartesian axes, *H*_*x*_ → *B*_1_, *H*_*y*_ → *B*_2_, and *H*_*z*_ → *A*_2_, but only the bare coordinate displacements of the oxygen atom transform as *C*_2*v*_ irreps, *O*_*x*_ → *B*_1_, *O*_*y*_ → *B*_2_, and *O*_*z*_ → *A*_1_. Symmetry-adapted linear combinations of the coordinate displacements of the hydrogen atoms transform as irreps, e.g., *H*_*1x*_ – *H*_2*x*_ → *A*_2_. These properties govern the pattern of vanishing values of the H_2_O AATs in Table 2, as well as those of the other basis sets reported in the SI.

**Table 2:** Electronic HF, MP2, and CID AATs (a.u.) for Water Using the cc-pVDZ Basis

	HF			MP2			CID		
	*B*_*x*_	*B*_*y*_	*B*_*z*_	*B*_*x*_	*B*_*y*_	*B*_*z*_	*B*_*x*_	*B*_*y*_	*B*_*z*_
*O*_1*x*_	–0.000000	–0.046076	0.000000	–0.000000	–0.047037	0.000000	–0.000000	–0.047906	0.000000
*O*_1*y*_	0.105707	–0.000000	0.000000	0.107082	–0.000000	0.000000	0.107556	–0.000000	0.000000
*O*_1*z*_	0.000000	0.000000	0.000000	–0.000000	0.000000	0.000000	–0.000000	0.000000	0.000000
*H*_2*x*_	0.000000	0.069789	–0.101645	0.000000	0.070693	–0.102471	0.000000	0.071165	–0.103160
*H*_2*y*_	–0.069867	0.000000	–0.000000	–0.070357	0.000000	–0.000000	–0.070850	0.000000	–0.000000
*H*_2*z*_	0.111684	–0.000000	–0.000000	0.112634	–0.000000	–0.000000	0.113396	–0.000000	–0.000000
*H*_3*x*_	–0.000000	0.069789	0.101645	–0.000000	0.070693	0.102471	–0.000000	0.071165	0.103160
*H*_3*y*_	–0.069867	–0.000000	0.000000	–0.070357	–0.000000	0.000000	–0.070850	–0.000000	0.000000
*H*_3*z*_	–0.111684	0.000000	–0.000000	–0.112634	0.000000	–0.000000	–0.113396	0.000000	–0.000000

The changes in the maximum and minimum correlated percentages are not as significant as that of the hydrogen molecule dimer. The minimum/maximum percentages associated with MP2 are 99.5/101.7, 99.3/101.5, 100.1/101.5, and 100.7/102.1% for STO-3G, 6-31G, 6-31G(d), and cc-pVDZ, respectively. For CID the minimum/maximum percentages are 99.3/104.6, 99.4/102.8, 101.0/102.6, and 101.4/104.0% for the same basis-set ordering. The largest gap between the minimum and maximum percentages occurs for CID/STO-3G at only 5.3%. Furthermore, the trend in differences between the minimum and maximum percentages are not nearly as consistent as the hydrogen molecule dimer test case. For MP2 the maximum/minimum percentage gap is 6-31G > STO-3G > 6-31G(d) > cc-pVDZ where the gap size ranges from 1.4 to 2.2%, whereas for CID, the gap size trend as STO-3G > 6-31G > cc-pVDZ > 6-31G(d) with the minimum value being 1.6%. For all the basis sets, the gap size is larger for CID compared to MP2.

The third test case, (*P*)-hydrogen peroxide, provides an optimized chiral system for which both AATs and VCD spectra may be examined. The AATs for (*P*)-hydrogen peroxide using the cc-pVDZ basis are provided in Table 3. (Again, the results from the remaining basis sets are provided in the SI). The minimum/maximum percentages for the MP2 method are 97.2/107.6, 89.0/104.4, 84.0/148.9, and 92.5/104.6% using the STO-3G, 6-31G, 6-31G(d) and cc-pVDZ basis sets, respectively. For CID, we observe similar changes to MP2 where the minimum/maximum percentages are 93.6/105.8, 91.0/102.7, 86.5/116.4, and 94.2/103.9% for the same basis sets, respectively. In contrast to both hydrogen molecule dimer and water, the largest difference between the minimum and maximum values (64.9%) was observed using the MP2 method with the 6-31G(d) basis set. However, similar to water, there is no clear trend between the basis set size and the minimum/maximum gap. For MP2, the gap size varies as 6-31G(d) > 6-31G > cc-pVDZ > STO-3G and for CID, 6-31G(d) > STO-3G > 6-31G > cc-pVDZ.

**Table 3:** Electronic HF, MP2, and CID AATs (a.u.) for (*P*)-Hydrogen Peroxide Using the cc-pVDZ Basis

	HF			MP2			CID		
	*B*_*x*_	*B*_*y*_	*B*_*z*_	*B*_*x*_	*B*_*y*_	*B*_*z*_	*B*_*x*_	*B*_*y*_	*B*_*z*_
*H*_1*x*_	0.004090	–0.032185	0.092323	0.004015	–0.031457	0.092030	0.004057	–0.032279	0.092993
*H*_1*y*_	0.056218	–0.089054	0.350998	0.056866	–0.093126	0.357087	0.057078	–0.092099	0.355837
*H*_1*z*_	–0.093657	–0.274700	0.085311	–0.094740	–0.277656	0.088809	–0.095085	–0.277008	0.087994
*H*_2*x*_	0.004090	–0.032185	–0.092323	0.004015	–0.031457	–0.092030	0.004057	–0.032279	–0.092993
*H*_2*y*_	0.056218	–0.089054	–0.350998	0.056866	–0.093126	–0.357087	0.057078	–0.092099	–0.355837
*H*_2*z*_	0.093657	0.274700	0.085311	0.094740	0.277656	0.088809	0.095085	0.277008	0.087994
*O*_3*x*_	–0.008638	0.065415	–0.109005	–0.008641	0.064745	–0.105808	–0.008649	0.065425	–0.106124
*O*_3*y*_	–0.014022	–0.046288	2.120337	–0.014337	–0.042809	2.113230	–0.014573	–0.043610	2.114382
*O*_3*z*_	0.063390	–2.049988	0.058282	0.064332	–2.046831	0.055502	0.064796	–2.048017	0.056045
*O*_4*x*_	–0.008638	0.065415	0.109005	–0.008641	0.064745	0.105808	–0.008649	0.065425	0.106124
*O*_4*y*_	–0.014022	–0.046288	–2.120337	–0.014337	–0.042809	–2.113230	–0.014573	–0.043610	–2.114382
*O*_4*z*_	–0.063390	2.049988	0.058282	–0.064332	2.046831	0.055502	–0.064796	2.048017	0.056045

### 4.2 Rotatory Strengths and Vibrational Circular Dichroism Spectra

**Figure 1:**
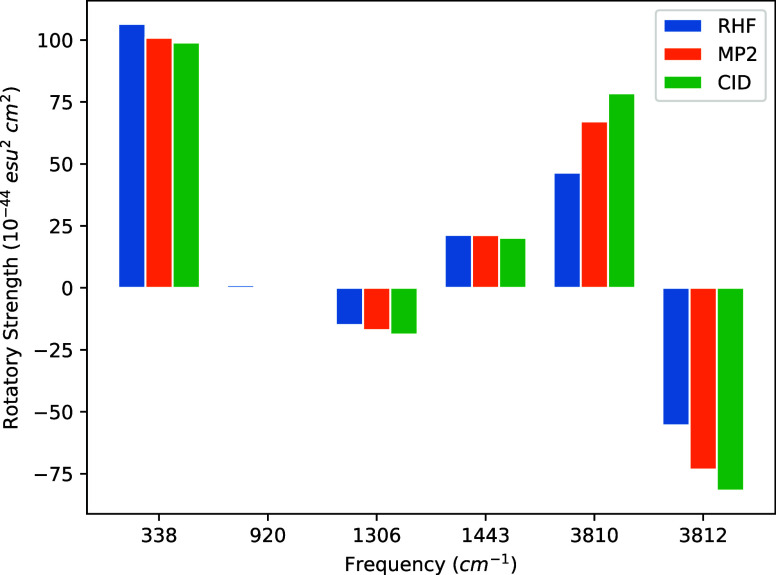
Bar graph of (*P*)-hydrogen peroxide rotational strengths using a common geometry and Hessian. The geometry and Hessian were computed using the cc-pVDZ basis at the MP2 level. The APTs and AATs were computed using the STO-3G basis set.

**Figure 2:**
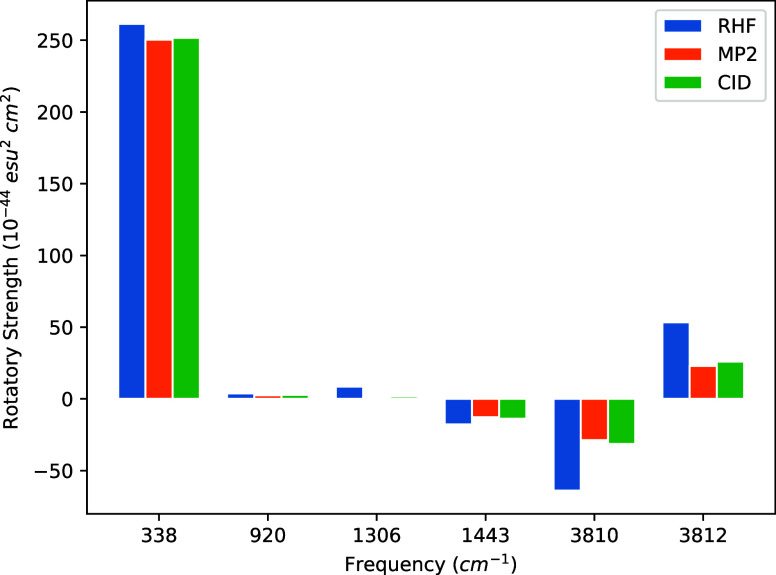
Bar graph of (*P*)-hydrogen peroxide rotational strengths using a common geometry and Hessian. The geometry and Hessian were computed using the cc-pVDZ basis at the MP2 level. The APTs and AATs were computed using the 6-31G basis set.

**Figure 3:**
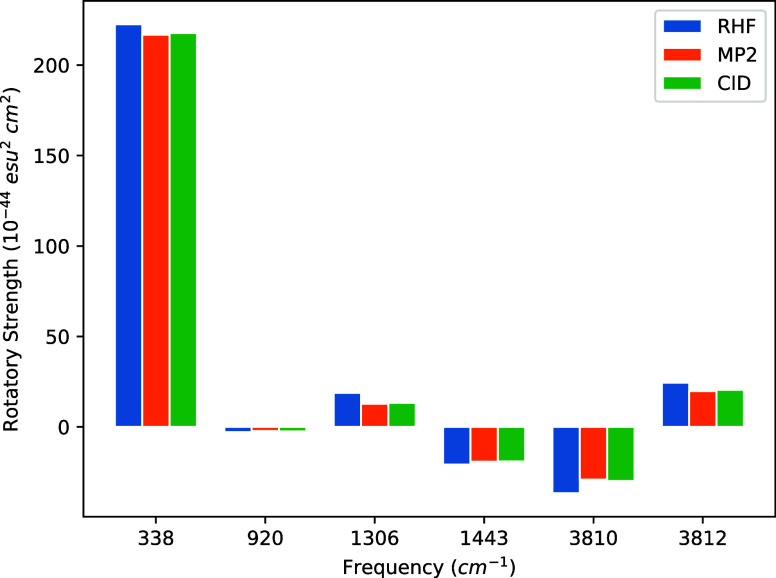
Bar graph of (*P*)-hydrogen peroxide rotational strengths using a common geometry and Hessian. The geometry and Hessian were computed using the cc-pVDZ basis at the MP2 level. The APTs and AATs were computed using the 6-31G(d) basis set.

**Figure 4:**
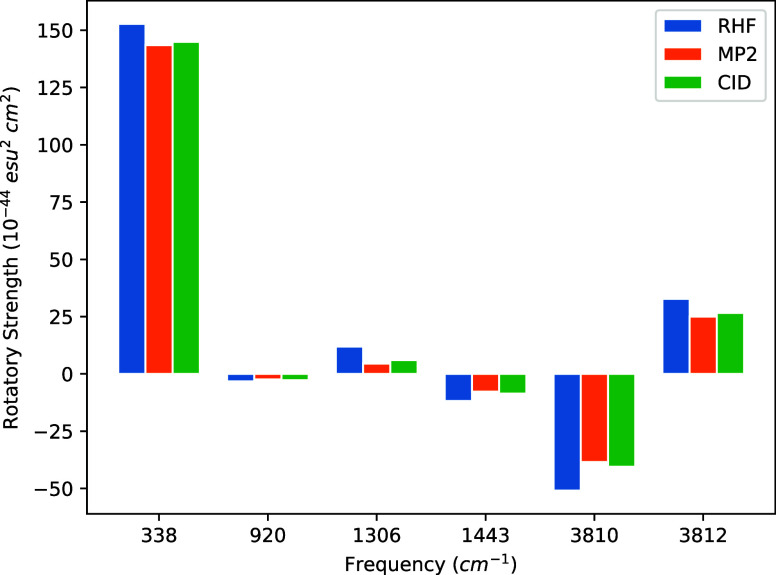
Bar graph of (*P*)-hydrogen peroxide rotational strengths using a common geometry and Hessian. The geometry and Hessian were computed using the cc-pVDZ basis at the MP2 level. The APTs and AATs were computed using the cc-pVDZ basis set.

The inclusion of dynamic electron correlation on the VCD spectra of (*P*)-hydrogen peroxide produces multiple effects, depending on the choice of basis set. The harmonic vibrational frequencies, IR intensities, and rotatory strengths of (*P*)-hydrogen peroxide for HF, MP2, and CID for the STO-3G, 6-31G, 6-31G(d), and cc-pVDZ basis sets are given in Tables 4, 5, 6 and 7. Additionally, for a more visual comparison, we include bar graphs of the rotatory strengths at each frequency in Figures 1, 2, 3, and 4. We note again that the same MP2/cc-pVDZ geometry and harmonic force constants were used for all levels of theory and basis sets in order to more clearly separate the impact of dynamic electron correlation on the VCD rotatory strengths from other effects, such as the optimized bond lengths and bond angles.

**Table 4:** Frequencies, IR Intensities, and Rotatory Strengths for (*P*)-Hydrogen Peroxide Using the STO-3G Basis^a^

	IR intensity (km/mol)			rotatory strength (10^–44^ esu^2^ cm^2^)		
frequency (cm^–1^)	HF	MP2	CID	HF	MP2	CID
3812.87	16.026	27.795	33.997	–55.443	–73.296	–81.774
3810.34	31.963	60.122	82.195	46.473	67.168	78.475
1443.26	1.213	1.198	1.113	21.365	21.260	20.191
1306.96	47.401	45.301	48.395	–14.985	–17.013	–18.822
920.51	0.068	0.013	0.029	1.071	0.460	0.660
338.53	134.248	118.958	113.545	106.476	100.907	98.960

a Quantities were obtained using a common MP2/cc-pVDZ geometry and Hessian.

**Table 5:** Frequencies, IR Intensities, and Rotatory Strengths for (*P*)-Hydrogen Peroxide Using the 6-31G Basis^a^

	IR intensity (km/mol)			rotatory strength (10^–44^ esu^2^ cm^2^)		
frequency (cm^–1^)	HF	MP2	CID	HF	MP2	CID
3812.87	15.191	2.533	3.309	53.349	22.911	26.009
3810.34	72.759	20.018	24.491	–63.938	–28.768	–31.378
1443.26	0.490	0.256	0.299	–17.797	–12.826	–13.823
1306.96	118.048	139.448	134.127	8.545	0.817	1.713
920.51	2.594	1.248	1.665	3.749	2.470	2.799
338.53	320.104	291.536	294.062	261.392	250.415	251.641

a Quantities were obtained using a common MP2/cc-pVDZ geometry and Hessian.

**Table 6:** Frequencies, IR Intensities, and Rotatory Strengths for (*P*)-Hydrogen Peroxide Using the 6-31G(d) Basis^a^

	IR Intensity (km/mol)			rotatory strength (10^–44^ esu^2^ cm^2^)		
frequency (cm^–1^)	HF	MP2	CID	HF	MP2	CID
3812.87	23.053	11.213	12.794	24.458	19.814	20.477
3810.34	94.143	50.167	56.910	–36.618	–29.131	–29.883
1443.26	0.478	0.411	0.406	–20.796	–19.249	–19.080
1306.96	120.017	136.651	131.984	18.794	12.799	13.311
920.51	2.745	1.690	2.044	–2.835	–2.254	–2.473
338.53	236.828	222.557	224.529	222.566	216.812	217.700

a Quantities were obtained using a common MP2/cc-pVDZ geometry and Hessian.

**Table 7:** Frequencies, IR Intensities, and Rotatory Strengths for (*P*)-Hydrogen Peroxide Using the cc-pVDZ Basis^a^

	IR Intensity (km/mol)			rotatory strength (10^–44^ esu^2^ cm^2^)		
frequency (cm^–1^)	HF	MP2	CID	HF	MP2	CID
3812.87	30.781	13.757	16.506	32.728	25.002	26.586
3810.34	117.644	57.086	67.839	–50.910	–38.440	–40.476
1443.26	0.246	0.106	0.132	–11.812	–7.731	–8.590
1306.96	105.238	114.319	110.611	11.921	4.499	6.029
920.51	2.456	1.292	1.683	–3.257	–2.396	–2.735
338.53	217.281	192.586	196.575	152.732	143.478	144.888

a Quantities were obtained using a common MP2/cc-pVDZ geometry and Hessian.

The most intense rotatory strengths across all basis sets correspond to the dihedral bending motion occurring at 338.53 cm^–1^. However, while this rotatory strength is the most prominent, its intensity, when introducing correlation effects, only deviates from that of HF by 2–7% across all basis sets. For the STO-3G basis, only the symmetric H–O–O bending transition at 1443.26 cm^–1^ exhibits smaller deviations from HF than the dihedral bend. For the 6-31G and cc-pVDZ sets, on the other hand, this transition shifts by 22–34%, whereas 6-31G(d) by only ∼8% for both MP2 and CID.

The next two most intense rotatory strengths correspond to the symmetric and antisymmetric hydrogen stretching modes at 3812.87 and 3810.34 cm^–1^. The former exhibits fairly strong correlation shifts relative to HF with STO-3G and 6-31G deviating between 32–57% and 6-31G(d) and cc-pVDZ deviating between 16–23%. Similarly, the antisymmetric O–H stretch changes significantly—by 69%—from HF at the CID/STO-3G level, a significant variation for this intense vibrational band. Using the STO-3G basis, the greatest deviation for MP2 is observed at 920.51 cm^–1^ for the oxygen stretching motion which changes by 57%, though this is also the weakest vibrational transition. For 6-31G, 6-31G(d), and cc-pVDZ, the rotatory strength most affected was the antisymmetric hydrogen bending motions observed at 1306.96 cm^–1^ for which deviations ranged between 29 and 90%.

**Figure 5:**
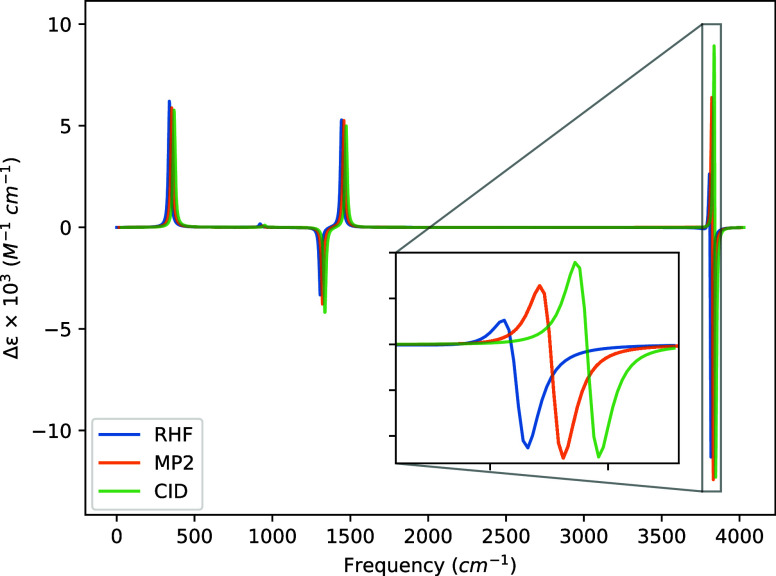
VCD spectra of (*P*)-hydrogen peroxide using a common geometry and Hessian. The geometry and Hessian were computed using the cc-pVDZ basis at the MP2 level. The APTs and AATs were computed using the STO-3G basis set. The full width at half-maximum was set to 16 cm^–1^. For readability, the MP2 and CID spectra were shifted by 15 and 30 cm^–1^, respectively.

**Figure 6:**
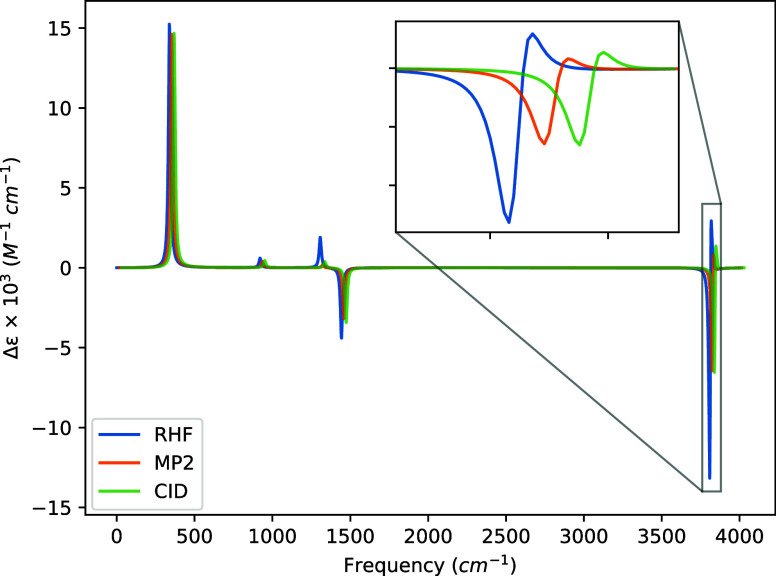
VCD spectra of (*P*)-hydrogen peroxide using a common geometry and Hessian. The geometry and Hessian were computed using the cc-pVDZ basis at the MP2 level. The APTs and AATs were computed using the 6-31G basis set. The full width at half-maximum was set to 16 cm^–1^. For readability, the MP2 and CID spectra were shifted by 15 and 30 cm^–1^, respectively.

**Figure 7:**
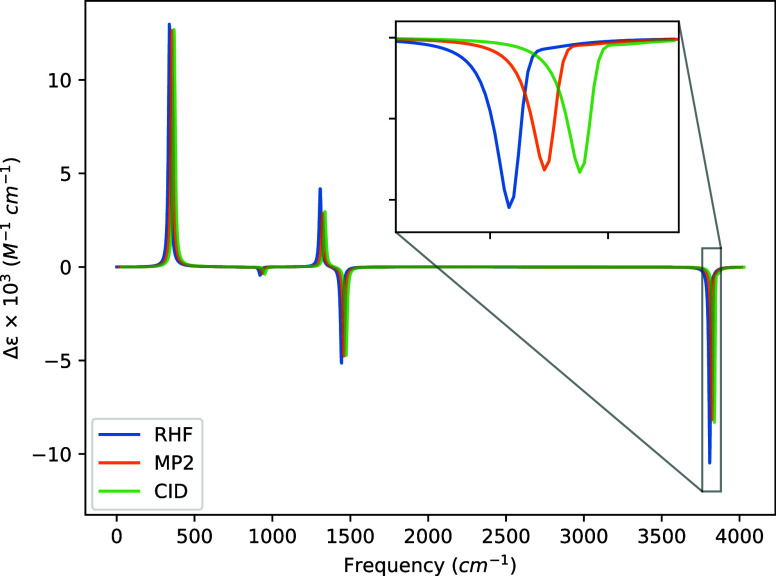
VCD spectra of (*P*)-hydrogen peroxide using a common geometry and Hessian. The geometry and Hessian were computed using the cc-pVDZ basis at the MP2 level. The APTs and AATs were computed using the 6-31G(d) basis set. The full width at half-maximum was set to 16 cm^–1^. For readability, the MP2 and CID spectra were shifted by 15 and 30 cm^–1^, respectively.

**Figure 8:**
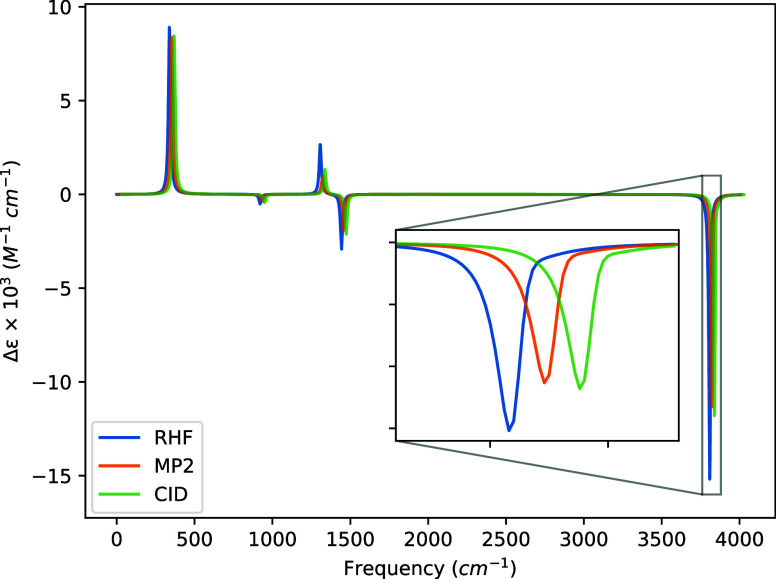
VCD spectra of (*P*)-hydrogen peroxide using a common geometry and Hessian. The geometry and Hessian were computed using the cc-pVDZ basis at the MP2 level. The APTs and AATs were computed using the cc-pVDZ basis set. The full width at half-maximum was set to 16 cm^–1^. For readability, the MP2 and CID spectra were shifted by 15 and 30 cm^–1^, respectively.

The VCD spectra generated from these data are presented in Figures 5, 6, 7, and 8, and we observe four notable features of these spectra across the various basis sets. First, the STO-3G basis exhibits changes in the signs of the rotatory strengths relative to the larger basis sets for all transitions above 1000 cm^–1^, even though the geometry and harmonic force constants used are identical in all cases. (We have confirmed this behavior at the HF level using the DALTON code.) Second, going from the 6-31G to the 6-31G(d) basis introduces a sign change for the transition at 920.51 cm^–1^, though, again, this is the weakest transition in the spectrum. Third, all three basis sets larger STO-3G tend to produce magnitudes of the rotatory strengths in the order of HF > CID > MP2 where the optimization of the CI coefficients corrects the MP2 transition intensity back toward that of the HF value. Finally, the symmetric hydrogen stretch at 3812.87 cm^–1^ becomes slightly washed out by the antisymmetric stretch at 3810.34 cm^–1^ as the basis set size increases and correlation effects are included. This is a direct result of our choice of full width at half-maximum, the near degeneracy of the two modes, and the difference in absolute rotatory strength between the symmetric and antisymmetric hydrogen stretching motions.

## 5 Conclusions

We have reported the first simulations of VCD spectroscopy including dynamic electron correlation using wave function methods, specifically the MP2 and CID levels of theory. Our implementation relies on a finite-difference scheme to obtain the Hessian, APTs, and AATs in the Cartesian coordinate basis. While the Hessian and APTs can be formulated as second derivatives of the energy, the AATs are formulated as overlaps between wave function derivatives due to the fact that the required electronic contributions to the magnetic-dipole transition moments are unphysically zero within the Born–Oppenheimer approximation. Subsequent transformation into the normal coordinate basis yields the vibrational frequencies, infrared intensities, and rotatory strengths required for simulating the absorption and VCD spectra.

We benchmarked our implementation using three small test cases including hydrogen molecule dimer, water, and (*P*)-hydrogen peroxide. The effects of correlation on the AATs are much more significant in the two chiral molecules than that of the achiral system (H_2_O) reaching a maximum deviation from the uncorrelated method of 21% (CID/cc-pVTZ) for hydrogen molecule dimer and 49% (MP2/6-31G(d)) for (*P*)-hydrogen peroxide while water only reaching a maximum deviation of 5% (CID/STO-3G). These effects appear concomitantly in the VCD spectra of (*P*)-hydrogen peroxide where the rotatory strength yields maximum deviations of 90% (MP2/6-31G). We note that five of the six transition intensities are of the incorrect sign for the small STO-3G basis set when compared to that of the cc-pVDZ set for every level of theory. Additionally, for the 6-31G, 6-31G(d), cc-pVDZ, we noted that optimization of the CI coefficients tends to correct the MP2 transition intensities back in the direction of the HF intensities with the maximum correction being approximately 13% (cc-pVDZ).

This work provides an avenue to benchmark future implementations of VCD using the MP2, CID, and higher levels of theory using analytic gradient methods. Due to the high computational scaling associated with computing determinants of nonorthogonal basis functions, future work will be directed at developing such analytic schemes.

## Supporting Information Available

Atomic coordinates; AATs; and rotatory strengths of the test molecules (PDF)
