# Supplementary material for: Simulation of Vibrational Circular Dichroism Spectra Using Second-Order Møller–Plesset Perturbation Theory and Configuration Interaction Doubles
Source: J Chem Theory Comput. 2024 Aug 13;20(16):7254–63. doi: 10.1021/acs.jctc.4c00747 (PMC11360133; doi:10.1021/acs.jctc.4c00747)
Supplement: Supplementary file 1 — ct4c00747_si_001.pdf [file ct4c00747_si_001.pdf]

Simulation of Vibrational Circular Dichroism Spectra Using Second-Order Møller-Plesset Perturbation Theory and Configuration Interaction Doubles

Brendan M. Shumberger and T. Daniel Crawford*

Department of Chemistry, Virginia Tech, Blacksburg, Virginia, U.S.A.

E-mail: crawdad@vt.edu

Contents

1 Geometries **2**

1.1 Hydrogen molecule dimer 2

1.2 Water 2

1.3 (P)-hydrogen peroxide 2

2 AAT Analysis **3**

2.1 Hydrogen molecule dimer 3

2.2 Water 4

2.3 (P)-hydrogen peroxide 5

3 VCD Analysis **7**

3.1 (P)-hydrogen peroxide 7

1 Geometries

1.1 Hydrogen molecule dimer

Table S1: Hydrogen molecule dimer geometry (a.u.)

Atom Type	Atom Number	X	Y	Z
H	1	-0.708647297046685	1.417294594093371	-0.613706561565607
H	2	0.	1.417294594093371	0.613706561565607
H	3	0.	-1.417294594093371	0.613706561565607
H	4	0.708647297046686	-1.417294594093371	-0.613706561565607

1.2 Water

Table S2: Water geometry (a.u.)

Atom Type	Atom Number	X	Y	Z
O	1	-0.000000000000000	0.000000000000000	0.128444410656440
H	2	0.000000000000000	-1.415531238764228	-1.019253001167221
H	3	0.000000000000000	1.415531238764228	-1.019253001167221

1.3 (P)-hydrogen peroxide

Table S3: (P)-hydrogen peroxide geometry (a.u.)

Atom Type	Atom Number	X	Y	Z
H	1	-1.780954530308296	1.411647335546379	0.872055376436941
H	2	1.780954530308296	-1.411647335546379	0.872055376436941
O	3	-1.371214332646589	-0.115525249760340	-0.054947416764017
O	4	1.371214332646589	0.115525249760340	-0.054947416764017

2 AAT Analysis

2.1 Hydrogen molecule dimer

Table S4: HF, MP2, and CID AATs (a.u.) for the hydrogen molecule dimer using the STO-3G basis.

	HF			MP2			CID		
	B_x	B_y	B_z	B_x	B_y	B_z	B_x	B_y	B_z
H_{1x}	-0.098393	-0.024606	0.069621	-0.097225	-0.024296	0.068761	-0.095541	-0.023838	0.067534
H_{1y}	0.025655	0.006121	-0.004117	0.025344	0.006049	-0.004075	0.024860	0.005931	-0.004023
H_{1z}	-0.210505	-0.052136	0.094229	-0.207874	-0.051506	0.093070	-0.203999	-0.050616	0.091451
H_{2x}	-0.088907	-0.022321	0.060429	-0.087874	-0.022075	0.059757	-0.086427	-0.021733	0.058841
H_{2y}	-0.017662	-0.004350	0.020878	-0.017401	-0.004307	0.020631	-0.016995	-0.004270	0.020306
H_{2z}	-0.216539	-0.053544	0.089290	-0.214000	-0.052882	0.088307	-0.210376	-0.051889	0.086924
H_{3x}	-0.088907	-0.022321	-0.060429	-0.087874	-0.022075	-0.059757	-0.086427	-0.021733	-0.058841
H_{3y}	-0.017662	-0.004350	-0.020878	-0.017401	-0.004307	-0.020631	-0.016995	-0.004270	-0.020306
H_{3z}	0.216539	0.053544	0.089290	0.214000	0.052882	0.088307	0.210376	0.051889	0.086924
H_{4x}	-0.098393	-0.024606	-0.069621	-0.097225	-0.024296	-0.068761	-0.095541	-0.023838	-0.067534
H_{4y}	0.025655	0.006121	0.004117	0.025344	0.006049	0.004075	0.024860	0.005931	0.004023
H_{4z}	0.210505	0.052136	0.094229	0.207874	0.051506	0.093070	0.203999	0.050616	0.091451

Table S5: HF, MP2, and CID AATs (a.u.) for the hydrogen molecule dimer using the 6-31G basis.

	HF			MP2			CID		
	B_x	B_y	B_z	B_x	B_y	B_z	B_x	B_y	B_z
H_{1x}	-0.151236	-0.038068	0.166493	-0.149933	-0.037638	0.165072	-0.148253	-0.036926	0.163116
H_{1y}	0.030195	0.006918	0.012945	0.030211	0.006944	0.012643	0.030093	0.006955	0.012330
H_{1z}	-0.418610	-0.102867	0.158222	-0.414723	-0.101974	0.156679	-0.409199	-0.100801	0.154702
H_{2x}	-0.127136	-0.032561	0.148738	-0.125798	-0.032316	0.147495	-0.124076	-0.032137	0.145864
H_{2y}	-0.015912	-0.004075	0.053462	-0.016058	-0.004172	0.053256	-0.016079	-0.004381	0.052888
H_{2z}	-0.408794	-0.100762	0.148849	-0.405368	-0.099829	0.147471	-0.400720	-0.098435	0.145733
H_{3x}	-0.127136	-0.032561	-0.148738	-0.125798	-0.032316	-0.147495	-0.124076	-0.032137	-0.145864
H_{3y}	-0.015912	-0.004075	-0.053462	-0.016058	-0.004172	-0.053256	-0.016079	-0.004381	-0.052888
H_{3z}	0.408794	0.100762	0.148849	0.405368	0.099829	0.147471	0.400720	0.098435	0.145733
H_{4x}	-0.151236	-0.038068	-0.166493	-0.149933	-0.037638	-0.165072	-0.148253	-0.036926	-0.163116
H_{4y}	0.030195	0.006918	-0.012945	0.030211	0.006944	-0.012643	0.030093	0.006955	-0.012330
H_{4z}	0.418610	0.102867	0.158222	0.414723	0.101974	0.156679	0.409199	0.100801	0.154702

Table S6: HF, MP2, and CID AATs (a.u.) for the hydrogen molecule dimer using the cc-pVDZ basis.

	HF			MP2			CID		
	B_x	B_y	B_z	B_x	B_y	B_z	B_x	B_y	B_z
H_{1x}	-0.078893	-0.008996	0.364298	-0.078875	-0.008600	0.362977	-0.078636	-0.007509	0.362176
H_{1y}	0.031734	0.009958	0.073418	0.031340	0.009951	0.073174	0.030302	0.009932	0.073471
H_{1z}	-0.529172	-0.138045	0.085432	-0.527501	-0.137850	0.085348	-0.525801	-0.138038	0.085000
H_{2x}	-0.053675	-0.025007	0.342479	-0.053706	-0.025413	0.341287	-0.053566	-0.026407	0.340754
H_{2y}	-0.024080	-0.008875	0.109311	-0.023638	-0.008864	0.108857	-0.022607	-0.008877	0.108197
H_{2z}	-0.523197	-0.123777	0.078075	-0.521843	-0.123201	0.078051	-0.520677	-0.122274	0.077854
H_{3x}	-0.053675	-0.025007	-0.342479	-0.053706	-0.025413	-0.341287	-0.053566	-0.026407	-0.340754
H_{3y}	-0.024080	-0.008875	-0.109311	-0.023638	-0.008864	-0.108857	-0.022607	-0.008877	-0.108197
H_{3z}	0.523197	0.123777	0.078075	0.521843	0.123201	0.078051	0.520677	0.122274	0.077854
H_{4x}	-0.078893	-0.008996	-0.364298	-0.078875	-0.008600	-0.362977	-0.078636	-0.007509	-0.362176
H_{4y}	0.031734	0.009958	-0.073418	0.031340	0.009951	-0.073174	0.030302	0.009932	-0.073471
H_{4z}	0.529172	0.138045	0.085432	0.527501	0.137850	0.085348	0.525801	0.138038	0.085000

Table S7: HF, MP2, and CID AATs (a.u.) for the hydrogen molecule dimer using the cc-pVTZ basis.

	HF			MP2			CID		
	B_x	B_y	B_z	B_x	B_y	B_z	B_x	B_y	B_z
H_{1x}	-0.032613	0.006533	0.473156	-0.032724	0.006935	0.472511	-0.032692	0.007925	0.471955
H_{1y}	0.024071	0.009098	0.109878	0.023585	0.009079	0.109889	0.022506	0.009046	0.110292
H_{1z}	-0.568261	-0.151186	0.034612	-0.567719	-0.151295	0.034726	-0.566818	-0.151653	0.034678
H_{2x}	-0.011180	-0.017924	0.450487	-0.011353	-0.018410	0.449966	-0.011487	-0.019431	0.449678
H_{2y}	-0.021442	-0.008826	0.134617	-0.020917	-0.008800	0.134254	-0.019845	-0.008774	0.133558
H_{2z}	-0.560927	-0.131426	0.026467	-0.560610	-0.131086	0.026648	-0.560127	-0.130365	0.026748
H_{3x}	-0.011180	-0.017924	-0.450487	-0.011353	-0.018410	-0.449966	-0.011487	-0.019431	-0.449678
H_{3y}	-0.021442	-0.008826	-0.134617	-0.020917	-0.008800	-0.134254	-0.019845	-0.008774	-0.133558
H_{3z}	0.560927	0.131426	0.026467	0.560610	0.131086	0.026648	0.560127	0.130365	0.026748
H_{4x}	-0.032613	0.006533	-0.473156	-0.032724	0.006935	-0.472511	-0.032692	0.007925	-0.471955
H_{4y}	0.024071	0.009098	-0.109878	0.023585	0.009079	-0.109889	0.022506	0.009046	-0.110292
H_{4z}	0.568261	0.151186	0.034612	0.567719	0.151295	0.034726	0.566818	0.151653	0.034678

2.2 Water

Table S8: HF, MP2, and CID AATs (a.u.) for water using the STO-3G basis.

	HF			MP2			CID		
	B_x	B_y	B_z	B_x	B_y	B_z	B_x	B_y	B_z
O_{1x}	0.000000	-0.179209	-0.000000	0.000000	-0.182230	-0.000000	0.000000	-0.187452	-0.000000
O_{1y}	0.199100	-0.000000	0.000000	0.198115	-0.000000	0.000000	0.197639	-0.000000	0.000000
O_{1z}	-0.000000	0.000000	-0.000000	-0.000000	0.000000	-0.000000	-0.000000	0.000000	-0.000000
H_{2x}	-0.000000	0.128114	-0.151198	-0.000000	0.129656	-0.152219	-0.000000	0.132318	-0.154568
H_{2y}	-0.043625	-0.000000	-0.000000	-0.043442	-0.000000	-0.000000	-0.043609	-0.000000	-0.000000
H_{2z}	0.093820	-0.000000	0.000000	0.094157	-0.000000	0.000000	0.095756	-0.000000	0.000000
H_{3x}	0.000000	0.128114	0.151198	0.000000	0.129656	0.152219	0.000000	0.132318	0.154568
H_{3y}	-0.043625	0.000000	0.000000	-0.043442	0.000000	0.000000	-0.043609	0.000000	0.000000
H_{3z}	-0.093820	0.000000	-0.000000	-0.094157	0.000000	-0.000000	-0.095756	0.000000	-0.000000

Table S9: HF, MP2, and CID AATs (a.u.) for water using the 6-31G basis.

	HF			MP2			CID		
	B_x	B_y	B_z	B_x	B_y	B_z	B_x	B_y	B_z
O_{1x}	-0.000000	-0.117585	-0.000000	0.000000	-0.117483	-0.000000	-0.000000	-0.118769	-0.000000
O_{1y}	0.205731	0.000000	-0.000000	0.204214	0.000000	-0.000000	0.204423	0.000000	-0.000000
O_{1z}	0.000000	0.000000	0.000000	0.000000	0.000000	0.000000	0.000000	0.000000	0.000000
H_{2x}	0.000000	0.048440	-0.072038	-0.000000	0.049167	-0.072617	0.000000	0.049793	-0.073420
H_{2y}	-0.035334	-0.000000	0.000000	-0.035465	0.000000	0.000000	-0.035835	-0.000000	0.000000
H_{2z}	0.071832	-0.000000	0.000000	0.072416	-0.000000	0.000000	0.073140	-0.000000	0.000000
H_{3x}	-0.000000	0.048440	0.072038	0.000000	0.049167	0.072617	-0.000000	0.049793	0.073420
H_{3y}	-0.035334	0.000000	-0.000000	-0.035465	0.000000	-0.000000	-0.035835	0.000000	-0.000000
H_{3z}	-0.071832	-0.000000	-0.000000	-0.072416	-0.000000	-0.000000	-0.073140	-0.000000	-0.000000

Table S10: HF, MP2, and CID AATs (a.u.) for water using the 6-31G(d) basis.

	HF			MP2			CID		
	B_x	B_y	B_z	B_x	B_y	B_z	B_x	B_y	B_z
O_{1x}	0.000000	-0.098572	0.000000	0.000000	-0.099480	0.000000	-0.000000	-0.100529	-0.000000
O_{1y}	0.142720	-0.000000	0.000000	0.143918	-0.000000	0.000000	0.144492	-0.000000	0.000000
O_{1z}	0.000000	-0.000000	-0.000000	0.000000	-0.000000	-0.000000	0.000000	-0.000000	-0.000000
H_{2x}	0.000000	0.047799	-0.071958	0.000000	0.048520	-0.072469	-0.000000	0.049055	-0.073187
H_{2y}	-0.043483	-0.000000	0.000000	-0.043530	-0.000000	0.000000	-0.043919	-0.000000	-0.000000
H_{2z}	0.071608	0.000000	-0.000000	0.072153	0.000000	-0.000000	0.072871	0.000000	-0.000000
H_{3x}	-0.000000	0.047799	0.071958	-0.000000	0.048520	0.072469	0.000000	0.049055	0.073187
H_{3y}	-0.043483	0.000000	0.000000	-0.043530	0.000000	0.000000	-0.043919	-0.000000	-0.000000
H_{3z}	-0.071608	-0.000000	0.000000	-0.072153	-0.000000	0.000000	-0.072871	-0.000000	0.000000

Table S11: HF, MP2, and CID AATs (a.u.) for water using the cc-pVDZ basis.

	HF			MP2			CID		
	B_x	B_y	B_z	B_x	B_y	B_z	B_x	B_y	B_z
O_{1x}	-0.000000	-0.046076	0.000000	-0.000000	-0.047037	0.000000	-0.000000	-0.047906	0.000000
O_{1y}	0.105707	-0.000000	0.000000	0.107082	-0.000000	0.000000	0.107556	-0.000000	0.000000
O_{1z}	0.000000	0.000000	0.000000	-0.000000	0.000000	0.000000	-0.000000	0.000000	0.000000
H_{2x}	0.000000	0.069789	-0.101645	0.000000	0.070693	-0.102471	0.000000	0.071165	-0.103160
H_{2y}	-0.069867	0.000000	-0.000000	-0.070357	0.000000	-0.000000	-0.070850	0.000000	-0.000000
H_{2z}	0.111684	-0.000000	-0.000000	0.112634	-0.000000	-0.000000	0.113396	-0.000000	-0.000000
H_{3x}	-0.000000	0.069789	0.101645	-0.000000	0.070693	0.102471	-0.000000	0.071165	0.103160
H_{3y}	-0.069867	-0.000000	0.000000	-0.070357	-0.000000	0.000000	-0.070850	-0.000000	0.000000
H_{3z}	-0.111684	0.000000	-0.000000	-0.112634	0.000000	-0.000000	-0.113396	0.000000	-0.000000

2.3 (P)-hydrogen peroxide

Table S12: HF, MP2, and CID AATs (a.u.) for (P)-hydrogen peroxide using the STO-3G basis.

	HF			MP2			CID		
	B_x	B_y	B_z	B_x	B_y	B_z	B_x	B_y	B_z
H_{1x}	-0.004484	0.008637	0.028702	-0.004520	0.009291	0.028081	-0.004613	0.008120	0.030381
H_{1y}	0.098229	-0.116025	0.304874	0.099327	-0.116441	0.304619	0.100952	-0.117457	0.305781
H_{1z}	-0.167187	-0.186584	0.129057	-0.169182	-0.186605	0.129414	-0.171975	-0.186835	0.130185
H_{2x}	-0.004484	0.008637	-0.028702	-0.004520	0.009291	-0.028081	-0.004613	0.008120	-0.030381
H_{2y}	0.098229	-0.116025	-0.304874	0.099327	-0.116441	-0.304619	0.100952	-0.117457	-0.305781
H_{2z}	0.167187	0.186584	0.129057	0.169182	0.186605	0.129414	0.171975	0.186835	0.130185
O_{3x}	-0.002565	0.108343	-0.178188	-0.002511	0.106186	-0.173263	-0.002401	0.106581	-0.167691
O_{3y}	-0.065450	-0.191618	0.644351	-0.066552	-0.188198	0.640964	-0.068167	-0.185026	0.640708
O_{3z}	0.138251	-0.526208	0.188112	0.141438	-0.528549	0.184699	0.145948	-0.537425	0.181882
O_{4x}	-0.002565	0.108343	0.178188	-0.002511	0.106186	0.173263	-0.002401	0.106581	0.167691
O_{4y}	-0.065450	-0.191618	-0.644351	-0.066552	-0.188198	-0.640964	-0.068167	-0.185026	-0.640708
O_{4z}	-0.138251	0.526208	0.188112	-0.141438	0.528549	0.184699	-0.145948	0.537425	0.181882

Table S13: HF, MP2, and CID AATs (a.u.) for (P)-hydrogen peroxide using the 6-31G basis.

	HF			MP2			CID		
	B_x	B_y	B_z	B_x	B_y	B_z	B_x	B_y	B_z
H_{1x}	0.006188	0.023353	0.007321	0.006085	0.024379	0.006512	0.006112	0.023651	0.007519
H_{1y}	0.038119	-0.150865	0.310585	0.038565	-0.154501	0.315174	0.038934	-0.154137	0.314618
H_{1z}	-0.068439	-0.163952	0.154625	-0.069351	-0.165654	0.157857	-0.069969	-0.165192	0.157521
H_{2x}	0.006188	0.023353	-0.007321	0.006085	0.024379	-0.006512	0.006112	0.023651	-0.007519
H_{2y}	0.038119	-0.150865	-0.310585	0.038565	-0.154501	-0.315174	0.038934	-0.154137	-0.314618
H_{2z}	0.068439	0.163952	0.154625	0.069351	0.165654	0.157857	0.069969	0.165192	0.157521
O_{3x}	-0.013836	0.116692	-0.156940	-0.013672	0.113221	-0.150905	-0.013649	0.113853	-0.149559
O_{3y}	-0.045036	-0.060622	1.165125	-0.044949	-0.055416	1.158389	-0.045382	-0.055186	1.159253
O_{3z}	0.100848	-1.088642	0.064540	0.101432	-1.088683	0.059810	0.102414	-1.090895	0.059515
O_{4x}	-0.013836	0.116692	0.156940	-0.013672	0.113221	0.150905	-0.013649	0.113853	0.149559
O_{4y}	-0.045036	-0.060622	-1.165125	-0.044949	-0.055416	-1.158389	-0.045382	-0.055186	-1.159253
O_{4z}	-0.100848	1.088642	0.064540	-0.101432	1.088683	0.059810	-0.102414	1.090895	0.059515

Table S14: HF, MP2, and CID AATs (a.u.) for (P)-hydrogen peroxide using the 6-31G(d) basis.

	HF			MP2			CID		
	B_x	B_y	B_z	B_x	B_y	B_z	B_x	B_y	B_z
H_{1x}	0.004682	0.002562	0.034734	0.004606	0.003815	0.033340	0.004630	0.002981	0.034406
H_{1y}	0.039117	-0.105749	0.312131	0.039577	-0.110083	0.317156	0.039850	-0.109219	0.316007
H_{1z}	-0.068464	-0.213244	0.105353	-0.069333	-0.214531	0.109395	-0.069764	-0.214024	0.108606
H_{2x}	0.004682	0.002562	-0.034734	0.004606	0.003815	-0.033340	0.004630	0.002981	-0.034406
H_{2y}	0.039117	-0.105749	-0.312131	0.039577	-0.110083	-0.317156	0.039850	-0.109219	-0.316007
H_{2z}	0.068464	0.213244	0.105353	0.069333	0.214531	0.109395	0.069764	0.214024	0.108606
O_{3x}	-0.007338	0.067388	-0.118570	-0.007360	0.066838	-0.115177	-0.007359	0.067554	-0.115664
O_{3y}	-0.037077	-0.019938	2.075807	-0.037359	-0.016740	2.064603	-0.037643	-0.017245	2.066379
O_{3z}	0.103477	-2.028530	0.024658	0.104485	-2.020605	0.021940	0.105036	-2.022912	0.022349
O_{4x}	-0.007338	0.067388	0.118570	-0.007360	0.066838	0.115177	-0.007359	0.067554	0.115664
O_{4y}	-0.037077	-0.019938	-2.075807	-0.037359	-0.016740	-2.064603	-0.037643	-0.017245	-2.066379
O_{4z}	-0.103477	2.028530	0.024658	-0.104485	2.020605	0.021940	-0.105036	2.022912	0.022349

Table S15: HF, MP2, and CID AATs (a.u.) for (P)-hydrogen peroxide using the cc-pVDZ basis.

	HF			MP2			CID		
	B_x	B_y	B_z	B_x	B_y	B_z	B_x	B_y	B_z
H_{1x}	0.004090	-0.032185	0.092323	0.004015	-0.031457	0.092030	0.004057	-0.032279	0.092993
H_{1y}	0.056218	-0.089054	0.350998	0.056866	-0.093126	0.357087	0.057078	-0.092099	0.355837
H_{1z}	-0.093657	-0.274700	0.085311	-0.094740	-0.277656	0.088809	-0.095085	-0.277008	0.087994
H_{2x}	0.004090	-0.032185	-0.092323	0.004015	-0.031457	-0.092030	0.004057	-0.032279	-0.092993
H_{2y}	0.056218	-0.089054	-0.350998	0.056866	-0.093126	-0.357087	0.057078	-0.092099	-0.355837
H_{2z}	0.093657	0.274700	0.085311	0.094740	0.277656	0.088809	0.095085	0.277008	0.087994
O_{3x}	-0.008638	0.065415	-0.109005	-0.008641	0.064745	-0.105808	-0.008649	0.065425	-0.106124
O_{3y}	-0.014022	-0.046288	2.120337	-0.014337	-0.042809	2.113230	-0.014573	-0.043610	2.114382
O_{3z}	0.063390	-2.049988	0.058282	0.064332	-2.046831	0.055502	0.064796	-2.048017	0.056045
O_{4x}	-0.008638	0.065415	0.109005	-0.008641	0.064745	0.105808	-0.008649	0.065425	0.106124
O_{4y}	-0.014022	-0.046288	-2.120337	-0.014337	-0.042809	-2.113230	-0.014573	-0.043610	-2.114382
O_{4z}	-0.063390	2.049988	0.058282	-0.064332	2.046831	0.055502	-0.064796	2.048017	0.056045

3 VCD Analysis

3.1 (P)-hydrogen peroxide

Table S16: Frequencies, IR intensities, and rotatory strengths for (P)-hydrogen peroxide using the STO-3G basis. Quantities were obtained using a common MP2/cc-pVDZ geometry and Hessian.

Frequency (cm^{-1})	IR Intensity (km/mol)			Rotatory Strength (10^{-44} esu^2 cm^2)		
	HF	MP2	CID	HF	MP2	CID
3812.87	16.026	27.795	33.997	-55.443	-73.296	-81.774
3810.34	31.963	60.122	82.195	46.473	67.168	78.475
1443.26	1.213	1.198	1.113	21.365	21.260	20.191
1306.96	47.401	45.301	48.395	-14.985	-17.013	-18.822
920.51	0.068	0.013	0.029	1.071	0.460	0.660
338.53	134.248	118.958	113.545	106.476	100.907	98.960

Table S17: Frequencies, IR intensities, and rotatory strengths for (P)-hydrogen peroxide using the 6-31G basis. Quantities were obtained using a common MP2/cc-pVDZ geometry and Hessian.

Frequency (cm^{-1})	IR Intensity (km/mol)			Rotatory Strength (10^{-44} esu^2 cm^2)		
	HF	MP2	CID	HF	MP2	CID
3812.87	15.191	2.533	3.309	53.349	22.911	26.009
3810.34	72.759	20.018	24.491	-63.938	-28.768	-31.378
1443.26	0.490	0.256	0.299	-17.797	-12.826	-13.823
1306.96	118.048	139.448	134.127	8.545	0.817	1.713
920.51	2.594	1.248	1.665	3.749	2.470	2.799
338.53	320.104	291.536	294.062	261.392	250.415	251.641

Table S18: Frequencies, IR intensities, and rotatory strengths for (P)-hydrogen peroxide using the 6-31G(d) basis. Quantities were obtained using a common MP2/cc-pVDZ geometry and Hessian.

Frequency (cm^{-1})	IR Intensity (km/mol)			Rotatory Strength (10^{-44} esu^2 cm^2)		
	HF	MP2	CID	HF	MP2	CID
3812.87	23.053	11.213	12.794	24.458	19.814	20.477
3810.34	94.143	50.167	56.910	-36.618	-29.131	-29.883
1443.26	0.478	0.411	0.406	-20.796	-19.249	-19.080
1306.96	120.017	136.651	131.984	18.794	12.799	13.311
920.51	2.745	1.690	2.044	-2.835	-2.254	-2.473
338.53	236.828	222.557	224.529	222.566	216.812	217.700

Table S19: Frequencies, IR intensities, and rotatory strengths for (P)-hydrogen peroxide using the cc-pVDZ basis. Quantities were obtained using a common MP2/cc-pVDZ geometry and Hessian.

Frequency (cm^{-1})	IR Intensity (km/mol)			Rotatory Strength (10^{-44} esu^2 cm^2)		
	HF	MP2	CID	HF	MP2	CID
3812.87	30.781	13.757	16.506	32.728	25.002	26.586
3810.34	117.644	57.086	67.839	-50.910	-38.440	-40.476
1443.26	0.246	0.106	0.132	-11.812	-7.731	-8.590
1306.96	105.238	114.319	110.611	11.921	4.499	6.029
920.51	2.456	1.292	1.683	-3.257	-2.396	-2.735
338.53	217.281	192.586	196.575	152.732	143.478	144.888
